# Time-dependent AC magnetometry and chain formation in magnetite: the influence of particle size, initial temperature and the shortening of the relaxation time by the applied field†

**DOI:** 10.1039/d1na00463h

**Published:** 2021-08-13

**Authors:** Irene Morales, Rocio Costo, Nicolas Mille, Julian Carrey, Antonio Hernando, Patricia de la Presa

**Affiliations:** Instituto de Magnetismo Aplicado (UCM-ADIF-CSIC) P.O. Box 155 Las Rozas Madrid 28230 Spain pmpresa@ucm.es; Instituto de Ciencia de Materiales de Madrid/CSIC Sor Juana Inés de la Cruz 3 Madrid 28049 Spain; Université de Toulouse, INSA, UPS, Laboratoire de Physique et Chimie des Nano-Objets (LPCNO), CNRS 135 Avenue de Rangueil, UMR 5215 F-31077 Toulouse France; Departamento de Física de Materiales, Universidad Complutense de Madrid Madrid 28048 Spain; Donostia International Physics Center 20018 Donostia Gipuzkoa Spain; IMDEA Nanociencia 28049 Madrid Spain; Universidad de Nebrija 28015 Madrid Spain

## Abstract

Magnetite nanoparticles (MNPs) with 12, 34 and 53 nm sizes have been measured by AC-magnetometry at 50 kHz and 57 mT maximum applied field. The MNPs form chains under the AC-field, and the dynamics of the formation can be studied by measuring hysteresis cycles at different times. The measurement time has been varied from 5 ms to 10 s and for different initial temperatures of 5, 25 and 50 °C. The chain formation, identified by the increase of susceptibility and remanence with the measurement time, appears only for 34 nm particles. It has been observed that saturation, remanence and susceptibility at low (high) fields increase (decrease) with time. For the other two samples, these magnitudes are independent of time. At low fields, the heating efficiency is higher at 5 °C than at 50 °C, whereas it shows an opposite behaviour at higher fields; the origin of this behaviour is discussed in the article. Additionally, the relaxation times, *τ*_N_ and *τ*_B_, have been calculated by considering the influence of the applied field. Chain formation requires translation and rotation of MNPs; therefore, the Brownian mechanism plays a fundamental role. It is found that magnetic reversal for 12 nm MNPs is mainly due to Néel relaxation. However, in the case of 34 nm MNPs, both mechanisms, Néel and Brownian relaxation, can be present depending on the amplitude of the field; for *μ*_0_*H* < 22 mT, the physical rotation of the particle is the dominant mechanism; on the other hand, for *μ*_0_*H* > 22 mT, both mechanisms are present within the size distribution. This highlights the importance of taking the field intensity into account to calculate relaxation times when analysing the relaxation mechanisms of magnetic colloids subjected to AC fields.

## Abstract 2

Dynamic of chains formation observed by time-dependent high frequency hysteresis loops.

## Graphical abstract

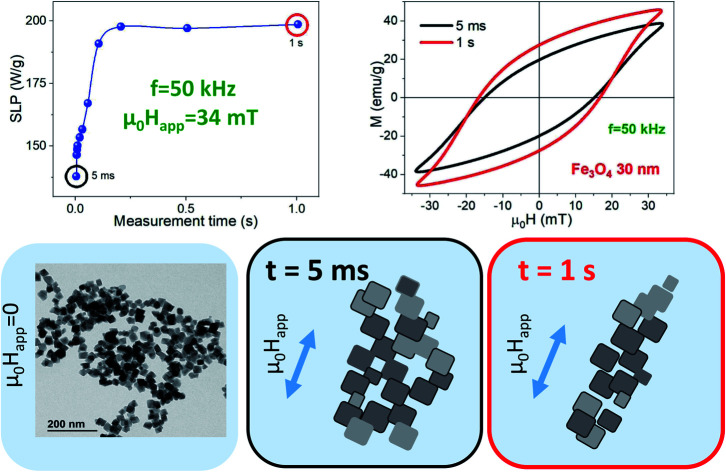

## 1. Introduction

Magnetic fluid hyperthermia is a well-known method that takes advantage of the heat released by magnetic nanoparticles when subjected to radiofrequency fields; the heat released causes cancer cell apoptosis or necrosis and, consequently, it is an appropriate method for the treatment of tumours.^1^ A deep comprehension of the influence of nanoparticle properties, colloidal media as well as the frequency and amplitude field range is relevant to optimize conditions for future successful clinical applications.^2–4^ AC-calorimetry is the most common technique for the determination of heating efficiency; however, the most recent AC magnetometry methods^5,6^ have been proven to be a very useful tool to obtain valuable information about the magnetic properties of dynamic systems, which are not accessible by calorimetric measurements. Parameters such as the coercive field, remanence or susceptibility can be determined from hysteresis loops, where the dependence on different variables (the applied field, frequency, MNP size, *etc.*) gives information about the magnetic interactions between particles, the possible arrangement of MNPs into self-organized assemblies, and the mechanisms of magnetic relaxation.^7–12^

Dipolar interactions are relevant when analysing the heating efficiency of colloidal MNPs as they influence magnetization dynamics substantially.^13^ Several theoretical and experimental studies show that dipole–dipole interactions can increase or decrease the heat released by MNPs depending on parameters such as anisotropy, the size and the concentration as well as the amplitude of the applied field when compared to the independent non-interacting case.^14–20^ Agglomeration caused by these long range and anisotropic interactions can favour ferromagnetic or antiferromagnetic alignment depending on the relative positions of MNP assemblies, which can cause (i) random agglomeration into pseudo spherical clusters, reducing their mobility and magnetic moment and leading to a magnetic flux closure configuration, which decreases their interaction with other agglomerates or (ii) anisotropic arrangements of MNPs into chains or columns which have been reported to increase the heating performance of colloids due to their enhanced uniaxial anisotropy.^7,21–27^

Asensio and coworkers^28^ were able to understand the origin of the differences in the heating performance of iron carbide nanoparticles (FeC) by measuring hysteresis cycles as a function of the measurement time. The authors reported that two samples of FeC with the same particle size and DC-magnetic properties but different ligand concentrations on the surface show different time dependence of the high frequency hysteresis cycles. The sample with the smallest agglomeration size shows high changes in susceptibility as well as in the shape of the magnetic cycles measured from 5 ms to 60 s, whereas the other one shows almost no time dependence. The authors attribute this behaviour to the formation of chains that are observed by TEM.^28^ More recently, Mille *et al.*^29^ reported chain formation during magnetic hyperthermia using time-dependent high-frequency hysteresis loops in 17 nm FeNi_3_ nanoparticles and observed that the chains are formed on a timescale ranging from several tens of seconds to less than 100 ms. This chain formation strongly depends on the magnetic field amplitude but does not depend on the frequency in the studied range (from 9 to 78 kHz).

In this work, we analyse the AC-hysteresis cycles of three magnetite samples with 12, 34 and 53 nm sizes, and present a thorough study on the heating efficiencies as a function of the MNP size, applied field, measurement time and initial sample temperature. It is observed that 34 nm MNPs are the only ones able to form chains, whereas chain formation is not observed in the other two samples. The explanation is that in the smallest one the main relaxation mechanism is Néel, and in the largest one, the high agglomeration size inhibits chain formation.

We also show the important role of the physical rotation of MNPs for arrangement into well-organized structures. Finally, in order to understand the differences observed between the samples, the evolution of the relaxation times is analysed as a function of the applied field for all the samples. It is found that the applied field shortens both Néel and Brownian relaxation times and, depending on the size distribution of the samples, both mechanisms can be present, which is observed for the MNPs with 34 nm. This is relevant for applications such as tumour treatment by hyperthermia using immobilized MNPs, catalytic reactions in solid matrices by inductive heating, *etc.*,^30,31^ since at high fields Brownian relaxation can jump to Néel relaxation, at least for certain particle size ranges.

## 2. Results and discussion

### 2.1 Structural characterization

**Fig. 1:**
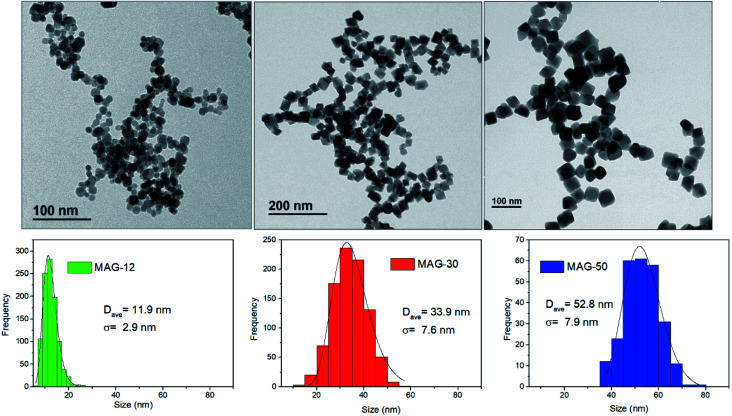
TEM images and size distribution fitted to a log normal function of the Fe_3_O_4_ nanoparticles.

The morphology and size distribution of MNPs are shown in Fig. 1. Sample MAG-12 shows a quasi-spherical shape, whereas samples MAG-30 and MAG-50 have an almost cubic shape. The histograms from the TEM images show a homogeneous MNP size distribution. XRD patterns are shown in Fig. S1,† with the diffraction peaks corresponding to an inverse spinel structure.

Samples MAG-12 and MAG-30 exhibit hydrodynamic sizes of 122 nm and 146 nm respectively, with a polydispersity index (PDI) below 0.2; however sample MAG-50 shows a *Z*-average of 2.6 μm and a PDI of 0.7, being much more polydisperse than the others and having a very broad size distribution with a huge agglomeration degree (Fig. S2†).

### 2.2 DC magnetic properties

Hysteresis cycles at 5 and 300 K and ZFC–FC curves at 10 mT are shown in Fig. S3.† The samples MAG-30 and MAG-50 exhibit a ferrimagnetic behaviour even at room temperature with saturation magnetization values (73 and 83 Am^2^ kg^−1^, respectively) close to the one reported for bulk Fe_3_O_4_ (see Table 2).^32,33^

The limit between single- and multi-domain nanoparticles, often called critical size *D*_c_, has been found to be around 80 nm, depending on the shape or anisotropy,^34–36^ and, according to this, we assume all the nanoparticles to be monodomain. The slight decrease of the coercive field for sample MAG-50 could be due to the oxidized external shell of the particles.

The ZFC–FC curves show that the MNPs are blocked at room temperature and seem to exhibit Verwey transition at about 50 K (see Fig. S3†), well below the 120 K of the bulk, typical for magnetite at the nanoscale.^37,38^ However, the kink in the ZFC observed for all the samples could also be attributed to other transitions, with the Verwey transition being depressed by the presence of the oxidized layer in the samples.

Sample MAG-12 has a *M*_r_/*M*_s_ value of 0.2 at 5 K, which means that a considerable amount of particles is blocked and randomly distributed, while at 300 K all the nanoparticles are superparamagnetic. Therefore, MAG-12 is formed by an assembly of single-domain NPs isotropically distributed. MAG-30 and MAG-50 also show remanence values below 0.33, indicating assemblies of random distributed nanoparticles.

The anisotropy field has been calculated at 5 K by considering the contribution of the thermal effects to the coercive field *H*_C_, especially relevant in the case of small particles, whose expression can be found elsewhere.^7,39^ The sample with the highest *H*_k_ is MAG-30 (75.1 mT) and the maximum applied field used in the AC magnetometric measurements (<60 mT) is smaller than *μ*_0_*H*_k_ for all the samples.

### 2.3 AC-magnetic properties

#### 2.3.1 Hysteresis cycles as a function of the applied field

**Fig. 2:**
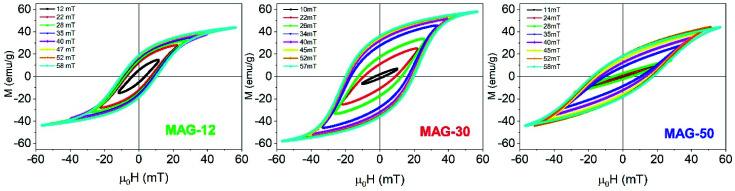
High frequency hysteresis cycles as a function of the applied field (*f* = 50.3 kHz) for MAG-12 (left), MAG-30 (center) and MAG-50 (right) for 10 s measurement time.

**Fig. 3:**
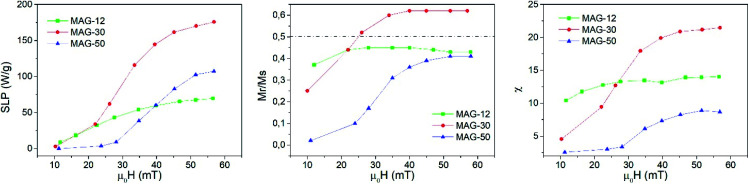
Evolution of the SLP (left), *M*_r_/*M*_s_ (center) and susceptibility (right) as a function of the applied field at 50.3 kHz and a measurement time of 10 s.

Fig. 2 shows the hysteresis cycles of colloidal suspensions as a function of the applied field (10–60 mT) at a frequency of 50.3 kHz and a measurement time of 10 s. By comparing all the samples, different behaviours are observed that depend on the intrinsic properties of the particles and on the applied field. It can be seen that, at low fields, sample MAG-12 has the highest area whereas at higher fields sample MAG-30 presents a squarer cycle with a larger area. As the area under the curves is the energy loss per cycle, these results shows that, at low fields, MAG-12 and MAG-30 have quite similar heating efficiencies, but the heating efficiency increases further for MAG-30 as the field increases (Fig. 3).

Remanence plays a significant role in the determination of the kinds of interactions. As is known, a remanence of 50% of saturation indicates a system of randomly oriented non-interacting MNPs; values below 50% reflect randomly oriented interacting nanoparticles, whereas values higher than 50% indicate anisotropic assemblies either because particles are oriented or the assemblies themselves are anisotropic, like in chain formation. In the case of MAG-12, the evolution of *M*_r_/*M*_s_ as a function of the field is almost constant around 40% (see Fig. 3). The *M*_r_/*M*_s_ value of sample MAG-50 increases from almost zero (the cycles are completely closed at 11 mT) to a value close to 40%, and saturated for fields around 50 mT. This indicates that both systems consist of randomly oriented interacting nanoparticles. However, as can be seen in Fig. 3, MAG-30 is the only one having remanence close to 60% for *μ*_0_*H* > 25 mT. This suggests the formation of an anisotropic assembly under the influence of the magnetic field, as already reported.^7,11,40^

Susceptibility, expressed as the slope of the hysteresis cycle at the coercive field, is shown in Fig. 3. MAG-12 shows a relatively high susceptibility (∼10–14) which remains almost independent of the applied field; the susceptibility of MAG-50 is low at low fields and then increases up to 8 at 57 mT. MAG-30 shows the highest susceptibility variation; it increases fivefold (from 4.5 to 21.5) in the whole field range. This high change of susceptibility together with the high remanence values confirms that chains can be formed for fields higher than 30 mT, as previously reported.^7,41^ It is worth noting that neither the smaller nor the larger particles show this huge change in susceptibility. In order to better understand the dynamics of these systems and the differences observed as a function of the size, a thorough study of the cycles as a function of the measurement time is shown in the next section.

#### 2.3.2 Hysteresis cycles as a function of the measurement time

Another unusual way to investigate the dynamic behaviour of MNPs subjected to alternating magnetic fields is to measure the cycles as a function of the measurement time at a given field.

**Fig. 4:**
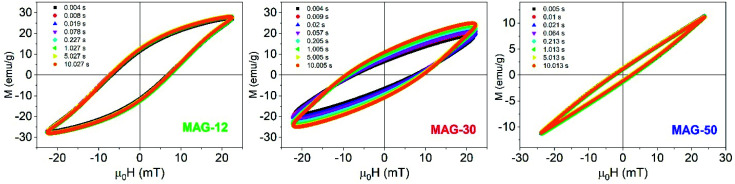
Evolution of the AC loops as a function of the measurement time for an applied field of 22 mT (*f* = 50.33 kHz).

Fig. 4 shows the hysteresis loops as a function of the measurement time for an applied field of 22 mT and at 50.3 kHz. It is observed that, in the case of MAG-30, magnetization, coercivity and susceptibility vary remarkably between 5 ms and 10 s whereas the other two samples show negligible changes.

**Fig. 5:**
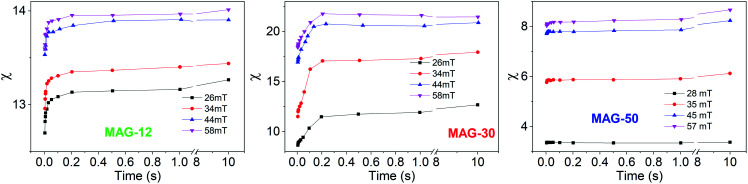
Susceptibility as a function of the measurement time for different applied fields.

To better understand this behaviour, Fig. 5 shows the susceptibility of all samples as a function of time from 5 ms to 10 s for different applied fields. In the time range from 5 to 200 ms, the susceptibility increases up to 50% at a low field and up to 15% at a high field for sample MAG-30. On the other hand, samples MAG-12 and MAG-50 show negligible changes with the measurement time, increments never exceeding 3% of the susceptibility at 5 ms.

**Fig. 6:**
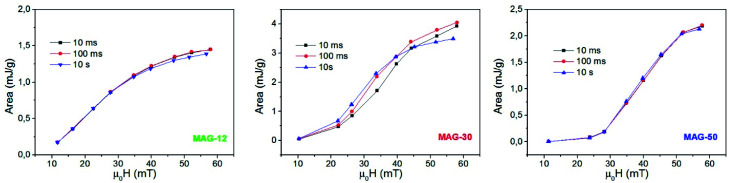
Evolution of the areas as a function of the applied field for different measurement times (*f* = 50.33 kHz).

Fig. 6 shows the evolution of the areas of the cycles as a function of the field for different times. The specific loss power (SLP) of samples MAG-12 and MAG-50 is independent of time. However, MAG-30 shows that at lower fields (*μ*_0_*H*_app_ < 40 mT), heating power increases with time, while at higher fields the area decreases after 100 ms. As can be seen in Fig. S4,† the magnetization of MAG-30 increases with time for an applied field of 22 mT but, at 57 mT, there is an initial increase and then it decreases. The same behaviour is observed for *M*_r_/*M*_s_ and *H*_c_. There are several possible interpretations of these results:

(i) At long times, the temperature increase of the sample would decrease the coercive field and saturation magnetization, leading to a smaller area of the hysteresis cycles;

(ii) The temperature increase would increase the entropy leading to a partial disorder of the particle chain, which would also produce a smaller hysteresis cycle.

(iii) A combination of the two previous interpretations.

In order to elucidate this point, measurements as a function of the initial temperature have been performed on sample MAG-30.

#### 2.3.3 Influence of the initial temperature on the magnetic behaviour

In order to understand the influence of the initial temperature (*T*_i_) of the system on the dynamic behaviour, the sample with the highest heating efficiency (MAG-30) was studied as a function of three different initial temperatures 5, 25 and 50 °C, for various magnetic fields and also as a function of the measurement time.

**Fig. 7:**
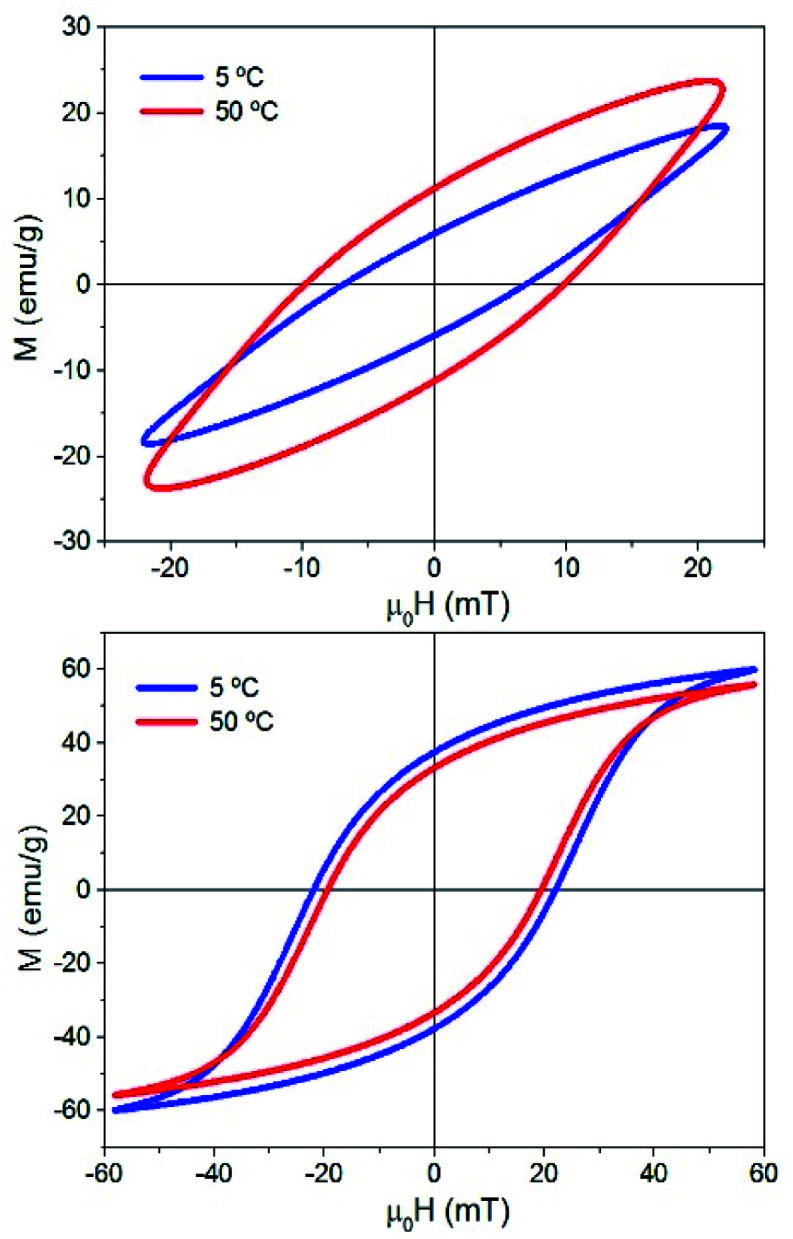
Hysteresis cycles of sample MAG-30 at *T*_i_ = 5 and 50 °C and 22 mT (above) and 57 mT (below). *t* = 10 s, *f*: 50.33 kHz.

**Fig. 8:**
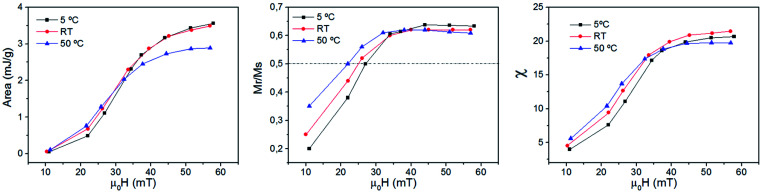
Area (left), *M*_r_/*M*_max_ (middle) and the susceptibility (right) as a function of the applied field for sample MAG-30 at different *T*_i_ values. The measurement time is 10 s.

Fig. S5 and S6† show all the hysteresis cycles at different *T*_i_ values and Fig. 7 shows only two hysteresis cycles at 5 and 50 °C, for applied fields of 22 mT and 57 mT at *t* = 10 s. Fig. 8 shows the area of the hysteresis cycles, the remanence *M*_r_/*M*_s_ and the susceptibility increase with the applied field for every *T*_i_.

**Fig. 9:**
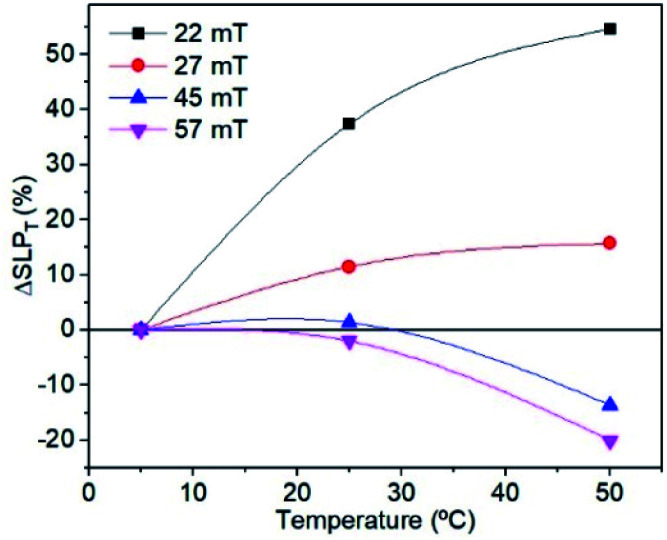
Evolution of the ΔSLP of sample MAG-30 as a function of *T*_i_ for different fields and *t* = 10 s.

It is possible to analyse the SLP increase with respect to the different *T*_i_ values and applied fields with the following equation:^8^1
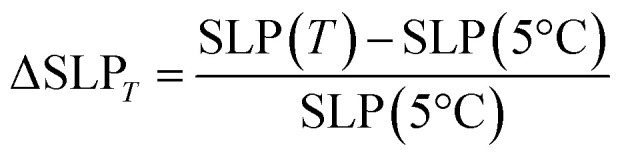
where ΔSLP_T_ is the total variation of the heating efficiency at a given *T*_i_ with respect to another fixed *T*_i_, SLP(*T*_i_) is the heating efficiency obtained at 25 °C or 50 °C and SLP(5 °C) is the one calculated at 5 °C. As can be seen in Fig. 9, the energy loss increases with increasing *T*_i_ for fields lower than 35 mT and decreases for higher fields. This effect has also been observed by other authors^8^ who reported that, for particles smaller than 13 nm, ΔSLP_*T*_ always decreases for increasing *T*_i_ independent of the applied field, but an exception appears for particles around 17 nm. In the latter case, they observed that ΔSLP_*T*_ increases for applied fields of 5 kA m^−1^ (∼7 mT) and then decreases for 15 kA m^−1^ (∼20 mT), similarl to the behaviour observed in the present work.

In order to understand this effect, it is necessary to discuss the effect of not only *T*_i_ but also the measurement time on the intrinsic properties of nanoparticles. Fig. 8 shows that the remanence at low fields is well below 50%, suggesting a system of randomly oriented interacting MNPs. As is known, the thermal energy decreases the media viscosity and allows a higher contribution of particles relaxed by Brownian relaxation. But this is only possible at relatively low fields, where the heat released by the MNPs is still low, chains have not yet been formed and Brownian relaxation is still dominant. In addition, the thermal energy also decreases the coercive field; when the applied field is below the coercive field, the SLP increases with temperature, but if the applied field is above the coercive field then SLP decreases.

Therefore, a possible origin of the decrease of ΔSLP_*T*_ is an increase of the thermal energy of the colloid that decreases the hysteresis area at high fields owing to the decrease of saturation, remanence, susceptibility and coercivity at high fields and high *T*_i_. (see Fig. 7 and 8), and also promoted by the additional role of the thermal energy against chain formation.

**Fig. 10:**
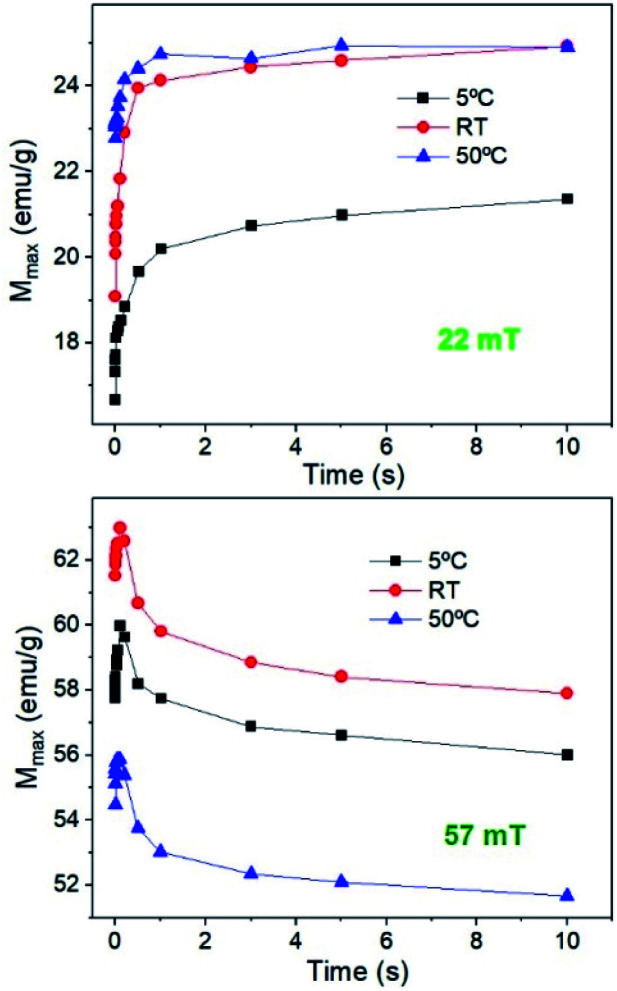
Evolution of the maximum M for sample MAG-30 at different *T*_i_ values as a function of time for 22 mT and 57 mT.

The hysteresis cycles at 22 mT, with different *T*_i_ values and measurement times show that the magnetization (as well as remanence, the coercive field and susceptibility) quickly increases up to 1 s and then remains almost constant independent of *T*_i_ (see Fig. 10). The behaviour is quite different for higher fields. Fig. 10 shows that, at 57 mT fields, an initial increase of *M* is observed up to around 200 ms for every *T*_i_, but then *M* decreases continuously with time. The hysteresis cycle at 57 mT (see Fig. S7†) shows a significant decrease of maximum *M* as the measurement time increases, but also a slight decrease of the remanence and *H*_c_ that leads to a decrease of the hysteresis area. The study of the hysteresis loops as a function of the measurement time reveals that, effectively, at high fields the system tends to form chains; however, as the time runs, the heat released by the nanoparticles, combined with the high *T*_i_, prevents chain formation and also leads to a decrease of *M*, *H*_c_, and *M*_r_/*M*_s_. It can be concluded that the SLP has strong dependence on the initial temperature of the sample; higher SLP values are obtained for higher *T*_i_ for fields below 35 mT, which is above the maximum field for biomedical applications; however smaller SLP values are obtained at higher *T*_i_ if the field is higher than 35 mT. This is important for application which requires high temperatures, because the temperature plays an opposite role at high fields.

The temperature increase of samples MAG-30 at 5 and 50 °C (Fig. S8†) has been measured simultaneously with hysteresis cycles to compare the SLP obtained by calorimetric and AC-magnetometric measurements under the exact same conditions.

It is already known that calorimetry characterization underestimates the SLP because of the lack of adiabatic conditions, especially at low particle concentrations or low applied fields.^42–44^

**Fig. 11:**
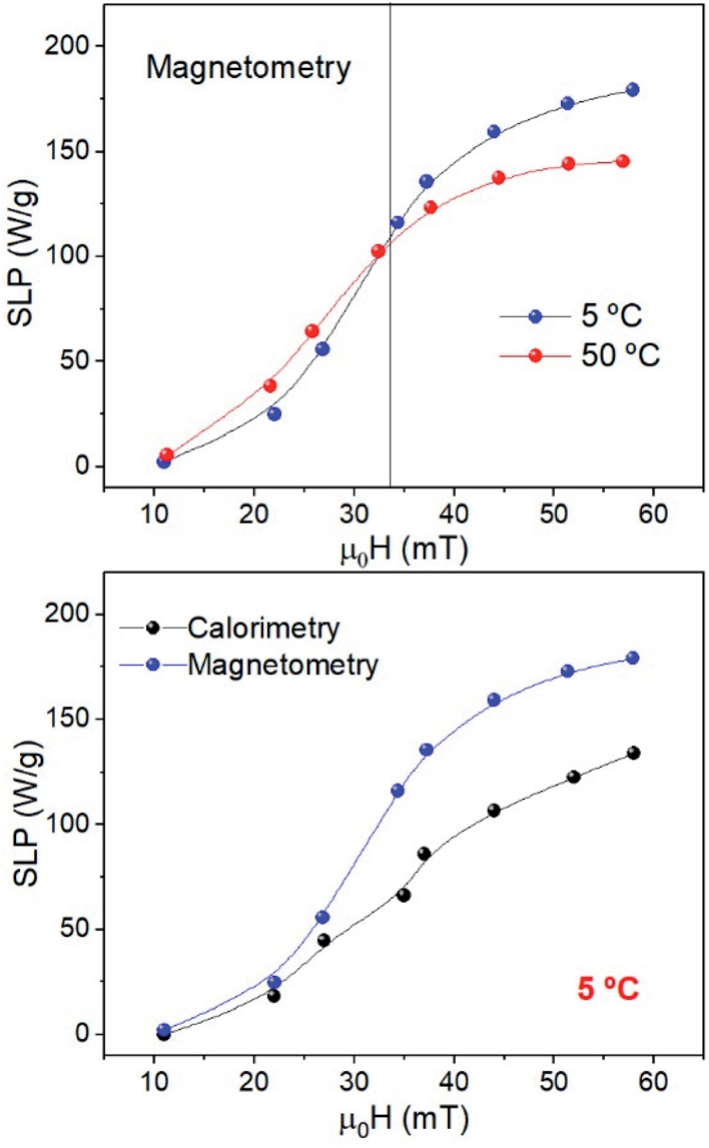
(Above) SLP *vs.* the field of sample MAG-30 obtained by AC-magnetometry at *T*_i_ = 5 °C and 50 °C (the black line is only to guide the eyes) and (below) comparison between calorimetry and AC-magnetometry for sample MAG-30 at *T*_i_ = 5 °C.

Our results show good qualitative agreement regarding the SLP behaviour for both methods (Fig. 11), but in the case of calorimetry, the values are underestimated by 30% especially at high fields, independent of *T*_i_. This happens when there are thermal losses or fast heating that causes large *T* gradients within the sample (making the measurement dependent on the position of the thermometer).^45,46^

### 2.4 Effect of the applied field on the relaxation time

When a magnetic field is applied, magnetization is reversed toward the field direction by means of two different mechanisms: (a) Néel rotation that corresponds to the reversal of the magnetic moment within the magnetic domain and (b) Brownian rotation in which the particle as a whole rotates towards the field due to the magnetic torque. The dominant relaxation mechanism depends on the ratio between the magnetic and thermal energy, and also on the applied field which decreases the energy barrier.^47^

Along this work, it has been proven that chain formation depends on the applied field, the higher the field is the more noticeable is the chain formation, either because there are more chains or because the chains are longer. This suggests that MNPs are moving in the colloid during the time the field is applied. It is worth noting that the mechanism that governs the translational and rotational motion of MNPs is the Brownian mechanism because the torque exerted by the field is transmitted to the MNPs. In most of the literature reports, the analysis on Brownian or Néel relaxation has been considered at zero applied field for different particle sizes and anisotropies. However, as the field decreases the energy barrier, a different scenario can be found. Therefore, in this section, we will discuss the effect of the applied field on the relaxation times to understand which is the dominant relaxation mechanism at a given field.

#### 2.4.1 Relaxation time at zero applied field

Briefly, the equilibrium relaxation times for both mechanisms, *τ*_N_ and *τ*_B_ can be determined respectively by means of eqn (2) and (3).2
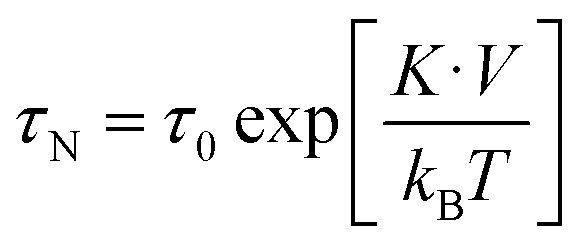
where *K* is the anisotropy constant, *V* the particle volume, *T* the temperature and *k*_B_ the Boltzmann constant. Note that for *K* = 0 the Néel relaxation time is *τ*_0_, in the order of 10^−9^ or 10^−10^ s.3
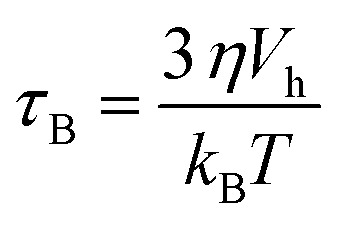


In the case of the Brownian relaxation time, *η* is the viscosity of the liquid and *V*_h_ stands for the nanoparticle hydrodynamic volume. When the anisotropy constant disappears, there is no nanoparticle rotation induced by the field, since in this case the magnetization is not linked to the nanoparticle lattice.

The calculated *τ*_N_ and *τ*_B_ at zero applied field are shown in Table S1.†

#### 2.4.2 Relaxation time at high fields

When a sufficient high external field is applied, as in this work, these equilibrium time constants can no longer precisely describe the dynamics. The magnetic moment of MNPs aligns faster than in the equilibrium case (when *H*_app_ = 0) and the timescales of the reversal and/or rotation are shorter than the equilibrium relaxation times. Moreover, the time to form chains also depends on the magnetic field gradient around the particles and theories and simulations must be used to discuss this rigorously.^48^

The Néel relaxation time as a function of the applied magnetic field can be estimated using eqn (4), which is an approximate expression calculated using the conventional Brown's equation,^47,49^ where *h* = *H*/*H*_k_.4
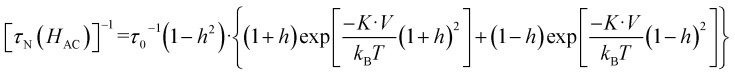


On the other hand, *τ*_B−eff_ can be calculated as a function of the applied field by the expression given by Yoshida *et al.*^50^ which also takes the rotation caused by the magnetic torque *μ*(*t*)·*H*(*t*) into account, which can be expressed as:5
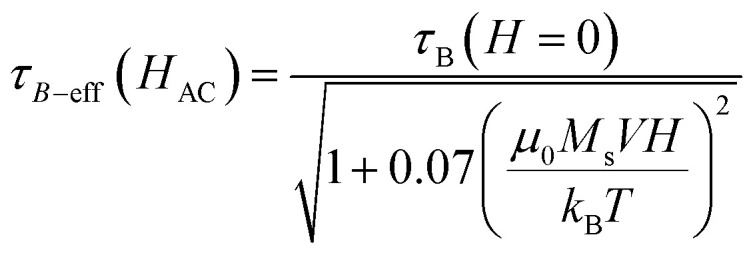


**Fig. 12:**
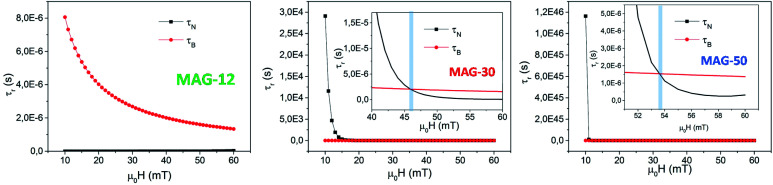
Evolution of the Brownian and Néel relaxation times as a function of the applied field according to eqn (4) and (5) for MAG-12 and MAG-30.

As can be seen, *τ*_B−eff_ (*H*_AC_) varies smoothly with the field and can be considered to be very similar to *τ*_B_ at zero applied field. The evolution of *τ* as a function of the field is shown in Fig. 12 for all the samples. For the calculations, the average particle size of each system, *K*_eff_ ≈ 10^4^ J m^−3^, *τ*_0_ = 10^−9^ s, a viscosity of *η* = 0.001 Pa s, *μ*_0_*H*_K_, *M*_s_ are considered from the SQUID measurements at 300 K and the hydrodynamic volume obtained from the DLS (Table 1).

**Table 1:** Sizes of the Fe_3_O_4_ samples obtained by TEM, XRD and DLS

Sample	TEM (nm)	XRD (nm)	Hydrodynamic size	
			*Z*-average (nm)	Mean number (nm)
MAG-12	11.9 ± 2.9	16.3	122 (0.20)	—
MAG-30	33.9 ± 7.6	27.5	146 (0.16)	95.3
MAG-50	52.8 ± 7.9	36.1	2638 (0.73)	848.3

**Table 2:** Magnetic properties of MNPs (SQUID measurements). Blocking temperatures *T*_B_ are all above 300 K. For the calculations of *μ*_0_*H*_K_: *f* = 10^−4^ Hz and *τ*_0_ = 10^−9^ s

Sample name	5 K				300 K		
	*μ*_0_ *H*_C_ (mT)	*μ*_0_ *H*_K_ (mT)	*M*_s_ (A m^2^ kg^−1^)	*M*_r_/*M*_s_	*μ*_0_ *H*_c_ (mT)	*M*_s_ (A m^2^ kg^−1^)	*M*_r_/*M*_s_
MAG-12	32.1	68.4	91.5	0.20	0.5	85.6	—
MAG-30	36.0	75.1	82.7	0.41	3.7	73.2	0.12
MAG-50	30.3	63.2	90.7	0.33	8.9	83.4	0.18

As can be seen, in the case of sample MAG-12, *τ*_N_ < *τ*_B_ for any field and the Néel relaxation dominates the magnetic reversal at any applied fields for all the sizes within the size distribution.

**Fig. 13:**
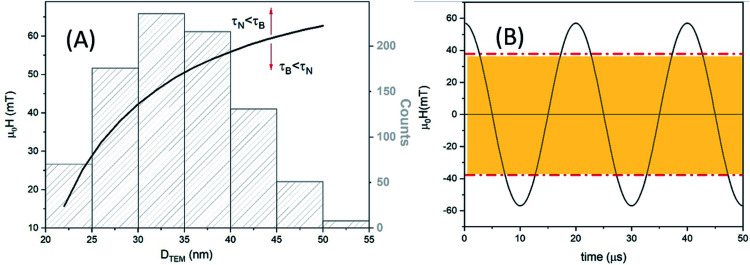
(A) Left axis: the black curve shows the applied field for which *τ*_N_ = *τ*_B_ as a function of particle size for MAG-30. Values below the curves correspond to *τ*_B_ < *τ*_N_, and values above one to *τ*_B_ > *τ*_N_. Right axis: MAG-30 particle size distribution. (B) For a particle with *d* = 28 nm and a maximum applied field of 57 mT, *τ*_B_ < *τ*_N_ during 50% of the field period (yellow zone), and *τ*_B_ > *τ*_N_ during the rest of time.

MAG-30 shows a quite different behaviour; it can change from Brownian relaxation at low fields to Néel relaxation at high fields. Taking the size distribution into account, the crossover between both mechanisms occurs between 16 and 62 mT (smallest–largest nanoparticles), as can be seen in the black curve in Fig. 13A, which shows the fields at which *τ*_N_ = *τ*_B_ as a function of the particle size. For fields below the curve, the dominant mechanism is the Brownian mechanism, and for field values above the curve, the reversal mechanism is by Néel.

However, both mechanisms can take place for the same MNPs depending on the applied field. For example, for the most frequent particle size (*d* = 34 nm), the Néel relaxation becomes dominant for fields higher than 45 mT; if the maximum applied field is 57 mT, Brownian relaxation takes place for |*μ*_0_*H*| < 45 mT, whereas the Néel magnetic reversal occurs for 45 mT < |*μ*_0_*H*| < 57 mT. Therefore, in a single field cycle, both mechanisms could be present in the same MNPs. Considering the sinusoidal variation of the field with a period of 20 μs, *τ*_B_ < *τ*_N_ during 80% of the field period, in a rough estimation. For particles with smaller sizes, for example 28 nm, *τ*_B_ < *τ*_N_ during 50% of the period (see Fig. 13B). As mentioned before, formation of chains is possible due to the physical rotation of the particles, *i.e.*, during the Brownian relaxation. During the time the particle relaxes by Néel relaxation, no chain formation occurs and the chain could be weakened by thermal fluctuations. On the other hand, at a low field (below 22 mT), the Brownian relaxation is the only mechanism present for all MNPs and the chain holds despite the increase of thermal energy. In the case of sample MAG-50, even if the Brownian relaxation mechanism is the dominant one for almost all the applied fields, the huge agglomeration of the MNPs prevents the formation of chains, as already reported.^28^

To sum up, according to these data it is possible to establish the following consideration about the magnetization curves:

(i) In the case of MAG-12, *τ*_N_ « *τ*_B_ independent of the applied field and the magnetic reversal is mainly by the Néel mechanism. The field shortens *τ*_N_, and *τ*_B_ remains several orders of magnitude above *τ*_N_, so there is neither rotation nor translation of the nanoparticles and no chains can be formed. In addition to that, smaller MNPs create a smaller dipolar field and the thermal fluctuations overcome the dipolar energy, not allowing the system to rearrange in the form of chains.

(ii) In the case of sample MAG-30, the mean MNP size shows *τ*_B_ < *τ*_N_ for *μ*_0_*H*_app_ < 45 mT and above this field, the Néel relaxation mechanism starts to dominate the reversal, in agreement with the experimental results obtained in this work. Therefore, both mechanisms can be present during a cycle of the field and the dominant one at each moment depends on the particle size and applied field. For a maximum applied field (57 mT) the Brownian relaxation takes place always at the smaller absolute values of the sinusoidal function, but as the values increase above the limiting curve of Fig. 13, the magnetization reverses by the Néel mechanism. The field at which this change takes places depends on the particle size, the larger the particles the more dominant is the Brownian relaxation. However, the onset of Néel relaxation is more frequent with increasing field, and the particle loses the translational rotation for a certain time during the field period.

(iii) In the case of sample MAG-50, the Brownian motion is the predominant one but there is no chain formation due to the agglomeration degree of the nanoparticles in the media. This highlights the fact that Brownian relaxation is a necessary but not sufficient condition for the chain formation to take place.

Even though the complexity of the mechanisms of chain formation cannot be completely and entirely explained by these considerations, these results reveal the importance of taking the field amplitude into account when calculating relaxation times to study the relaxation mechanisms of MNPs in colloids.

## 3. Conclusions

AC-magnetometric characterization of magnetite nanoparticles with 12, 34 and 52 nm particle sizes has been performed considering different variables: (i) the applied field amplitude, (ii) the measurement time and (iii) the initial temperature of the sample.

It can be concluded that the increase of susceptibility together with the *M*_r_/*M*_s_ ratio above 0.5 is the footprint for chain formation, and only the 34 nm MNPs showed this characteristic. These particles also show strong dependence of the hysteresis cycles on time; the magnetization, coercivity, remanence and susceptibility can increase or decrease with the measurement time depending on the amplitude of the applied field: for *μ*_0_*H*_app_ < 40 mT, the SLP increases with the time; however, for higher fields, magnetization, coercivity, remanence and susceptibility, and consequently the heating efficiency, decrease for long times.

The influence of the initial temperature on the heating efficiency has been investigated for sample MAG30. It is found that, at low fields, the most efficient system is the one with the highest initial temperature and, at higher fields, the behaviour is the opposite. This effect is dominated by the relation between the applied field and the coercive field. When the applied field is smaller than the coercive field, the high temperature decreases the media viscosity and the hysteresis cycles open, resulting in a higher SLP. If the applied field is higher than the coercive field, the high initial temperature, added to the high temperature increase induced by the applied field, leads to the decrease of magnetization, coercivity and remanence and the decrease of the SLP.

Regarding the relaxation mechanisms, both Néel and Brown relaxation times shorten when a magnetic field is applied because the energy barrier is decreased by the Zeeman energy; but Néel relaxation time decreases faster than the Brownian one. The chain formation depends on the applied field, *i.e.*, the rotation and translation of MNPs are required to form the chains. The mechanism that allows the translations and rotations of MNPs is the Brownian one; therefore, it is important to determine how much the Néel relaxation time is reduced by the applied field. Sample MAG-12 shows Néel relaxation, independent of the applied field, so there is no chain formation. However, the MAG-30 sample shows a different behaviour; large particles that have Brownian relaxation at zero (or very low) applied field changes to Néel relaxation due to the high applied field. Moreover, according to the calculations, the particles could suffer both mechanisms in a single cycle: Brownian relaxation during the part of the cycle with low values of the field, and Néel relaxation in the rest of the period. Further models are required to determine if the coexistence of both mechanisms in a single cycle is possible.

## 4. Methods

### 4.1 Synthesis

The sample with the smallest MNP size (MAG-12) has been synthetized by the well-known co-precipitation method, which is described elsewhere.^51,52^ Larger magnetite nanoparticles (MAG-30 and MAG-50) have been synthetized by the procedure known as oxidative precipitation.^53,54^ In this method, an Fe(ii) salt aqueous solution (water/ethanol) is precipitated in basic media in the presence of a mild oxidant, using a three-necked round flask under mechanical stirring in a nitrogen atmosphere. Changing the iron salt precursor, the oxidant and the quantity of ethanol in the reaction, it is possible to obtain magnetite nanocrystals with sizes ranging from 20–60 nm and different degrees of polydispersity, shape and internal structure.

In this study, FeSO_4_ precipitation and subsequent ageing were carried out in a glove-box to avoid oxidation of the particles. The synthesis reactor is a 2 L jacketed baker thermostatized to 90 °C. Two solutions were prepared independently outside the reactor: (a) 50 mL of FeSO_4_·7H_2_O at 0.2 M dissolved in 10^−2^ M H_2_SO_4_ and (b) 200 mL of water containing NaOH and KNO_3_ to obtain the final concentration of 0.2 M NaNO_3_ and 0.422 M or 0.46 M NaOH (for samples MAG-30 and MAG-50, respectively). In the case of sample MAG-30, 25% EtOH (96%) was also added to the solution. Then, iron(ii) sulfate solution was added to the basic solution at a constant rate and under stirring. A green rust dispersion was then formed.

This green rust dispersion was mechanically stirred for another 30 minutes and transferred to the thermostatized reactor, where the system was left undisturbed and heated to 90 °C for 24 h. The ageing time was fixed at 24 h in order to reach conditions near equilibrium. At this point, the solution was cooled down to room temperature using an ice bath, and the solid was separated by magnetic decantation and washed several times with distilled water.^51,53,55^

A standard protocol was used to oxidize the particle surface from magnetite to maghemite (γ-Fe_2_O_3_), and to activate the particle surface.^56^ Briefly, 300 mL of HNO_3_ (2 M) was added to the particles, and the mixture was stirred for 15 min. Then, nitric acid was removed by magnetic decantation, and 75 mL of Fe(NO_3_)_3_ (1 M) and 130 mL of water were added to the particles. The mixture was heated up to the boiling temperature and stirred for 30 min. The particles were then cooled to room temperature; the supernatant was substituted by 300 mL of HNO_3_ (2 M) by magnetic decantation and stirred for 15 min. Finally, the particles were washed three times with acetone and redispersed in water. A rotary evaporator was used to remove any acetone waste and concentrate the sample. This acid treatment enhances the colloidal properties of the samples by charging the nanoparticle surface, therefore making them more stable in the aqueous dispersion. It also causes a controlled surface oxidation of the magnetite into maghemite phase, making them more stable and biocompatible. In addition, in the case of smaller nanoparticles, the treatment recrystallizes the surface and increases the structural order, improving their magnetic properties.^54,56^

### 4.2 Structural and colloidal characterization

The crystal structures of the samples were identified by X-ray powder diffraction on a Panalytical X'Pert MPD using Cu K_α_ radiation; the patterns were collected at 2*θ* from 10° to 90°with steps of 0.04°. Transmission Electron Microscopy (TEM) was performed using a JEOL JEM1010 operating at 100 keV to determine the size, shape and distribution of the MNPs. To this end, one drop of a dilute suspension of the MNPs was deposited on a copper grid covered with a thin carbon film and allowed to evaporate at room temperature. For the determination of the mean size and distribution, at least 300 particles were analysed measuring their longest length and fitted to a log normal distribution. The value of the hydrodynamic size of the samples has been measured in a Zetasizer Nano ZS Malvern, using the average value of three consecutive measurements for each sample. The samples were analysed in water at 25 °C. The iron concentration was determined using an Inductively Coupled Plasma-Optical Emission Spectrometer (ICP-OES) by digesting a known volume of sample in a mixture of nitric and hydrochloric acid.

### 4.3 DC-magnetic characterization

DC-magnetic characterization was carried out in a SQUID Quantum Design MPMS-5S magnetometer. The measurements were performed in powder form in a glycerine sample holder. Magnetization *vs.* field curves at 5 T were measured at 5 and 300 K as well as the zero field-cooled (ZFC) and field cooled (FC) curves were measured at 10 mT between 5 and 300 K.

### 4.4 AC-magnetic and calorimetric characterization

High-frequency hysteresis loops were measured in a home-made setup,^5^ placing 0.5 mL of sample in a 6 mm diameter sample holder. The magnetic field frequency was 50 kHz with the amplitude ranging from 10 to 60 mT. In addition, the time evolution of the hysteresis loops was characterized by extracting the data of the cycles at different measurement times, from 5 ms to 10 s. The sample concentration was around 30 mg mL^−1^ for samples MAG-12 and MAG-30, and around 50 mg mL^−1^ for sample MAG-50. The specific loss power was calculated as the product of the hysteresis area (*A*) and the field frequency (*f*): SLP = *Af*.

Calorimetric characterization was performed in the same homemade hysteresis loop meter measuring the temperature increase simultaneously with the hysteresis loop using two fiber optic thermometers (Reflex 4, Neoptix) set at different sample heights. The temperature rise of the samples was measured starting at different initial temperatures (5, 25 and 50 °C). The average of the two thermometers has been taken into account for calculating SLP, by means of the following equation:6
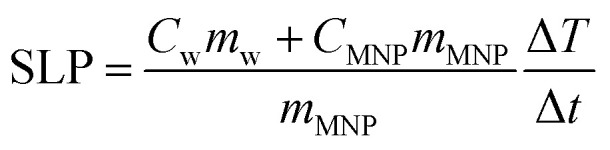
where *C*_w_ is the specific heat capacity of the dispersion media (4.185 J gK^−1^), *m*_w_ is the water mass, *m*_MNP_ is the MNP mass and 
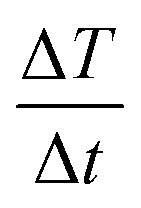
 is the maximum temperature slope in the first few seconds after turning the magnetic field on. As the mass of the MNPs is much smaller than the mass of water, the second term in the numerator can be disregarded.^37^

## Funding sources

This research was funded by Ministerio de Economía y Competitividad (MINECO) grant number RTI2018-095856-B-C21, Madrid Region grant number S2018/NMT-4381-MAT4.0-CM.C and 7th framework European project Nanomag 604448.

## Author contributions

Investigation and resources, I. M., R. C and N. M.; data curation and analysis, I. M, P. d. l. P, A. H.; visualization and supervision, J. C, P. d. l. P; writing-original draft, I. M, P. d. l. P, A. H.; writing-review & editing, all authors; project administration and funding, P. d. l. P.

## Conflicts of interest

The authors declare no competing financial or non-financial interest.

## Supplementary Material

NA-003-D1NA00463H-s001
