# Supplementary material for: Time-dependent AC magnetometry and chain formation in magnetite: the influence of particle size, initial temperature and the shortening of the relaxation time by the applied field
Source: Nanoscale Adv. 2021 Aug 13;3(20):5801–12. doi: 10.1039/d1na00463h (PMC9417483; doi:10.1039/d1na00463h)
Supplement: NA-003-D1NA00463H-s001 [file NA-003-D1NA00463H-s001.pdf]

Electronic Supplementary Material (ESI) for Nanoscale Advances.
This journal is © The Royal Society of Chemistry 2021

Electronic Supplementary Material (ESI) for Nanoscale Advances.

SUPPLEMENTARY INFORMATION: Time-dependent AC magnetometry and chain formation in magnetite: the influence of particle size, initial temperature and the shortening of the relaxation time by the applied field.

Irene Morales,[a] Rocio Costo,[b] Nicolas Mille,[c] Julian Carrey,[c] Antonio Hernando,[a,d,e] and Patricia de la Presa[*,a,d]

[a] *Instituto de Magnetismo Aplicado (UCM-ADIF-CSIC), P.O. Box 155, Las Rozas, Madrid 28230, Spain.*
[b] *Instituto de Ciencia de Materiales de Madrid/CSIC, Sor Juana Inés de la Cruz 3, Madrid 28049, Spain*
[c] *Universite´ de Toulouse, INSA, UPS, Laboratoire de Physique et Chimie des Nano-Objets (LPCNO), 135 Avenue de Rangueil, F-31077 Toulouse, France and CNRS, UMR 5215, LPCNO, F-31077 Toulouse, France.*
[d] *Departamento de Física de Materiales, Universidad Complutense de Madrid, Madrid 28048, Spain.*
[e] *Donostia International Physics Center, 20018, Donostia, Gipuzkoa; IMDEA Nanociencia, 28049, Madrid; Universidad de Nebrija, 28015, Madrid, Spain*
* Corresponding author: pmpresa@ucm.es

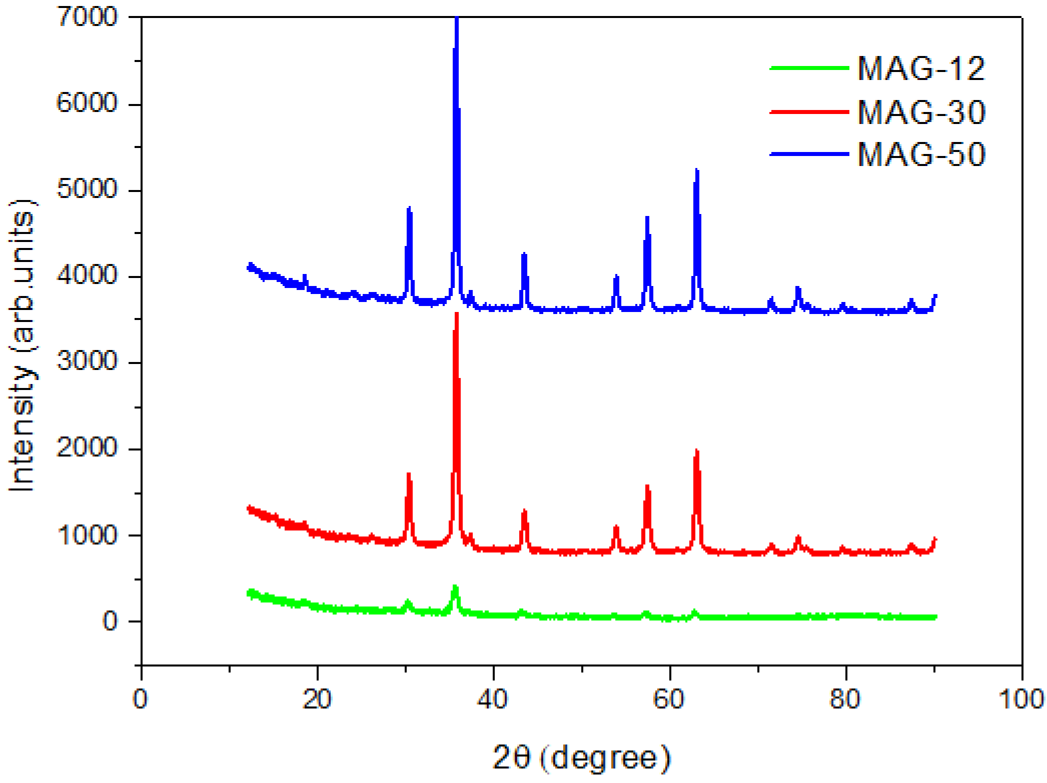

Fig. S1. XRD patterns of samples MAG-12, MAG-30 and MAG-50.

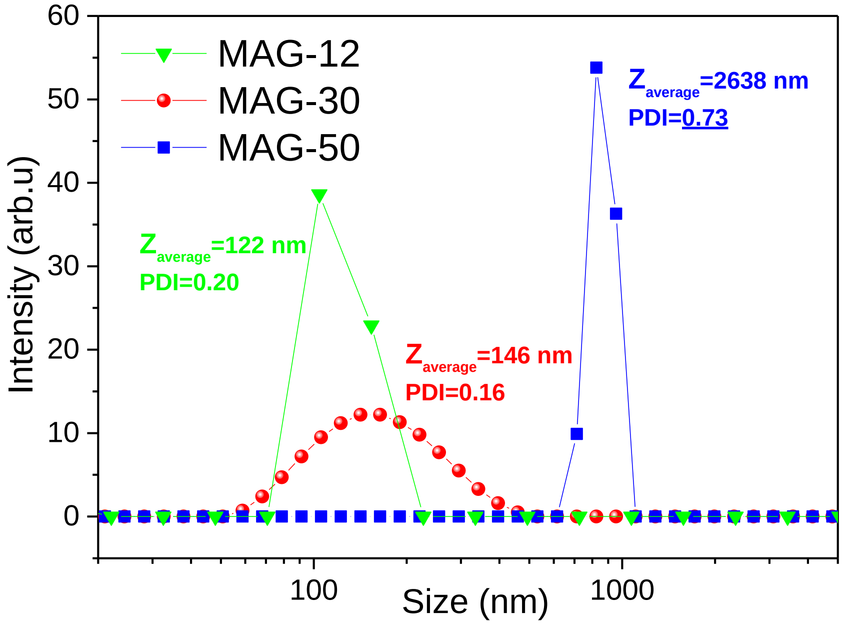

Fig.S2. DLS Intensity measurements.

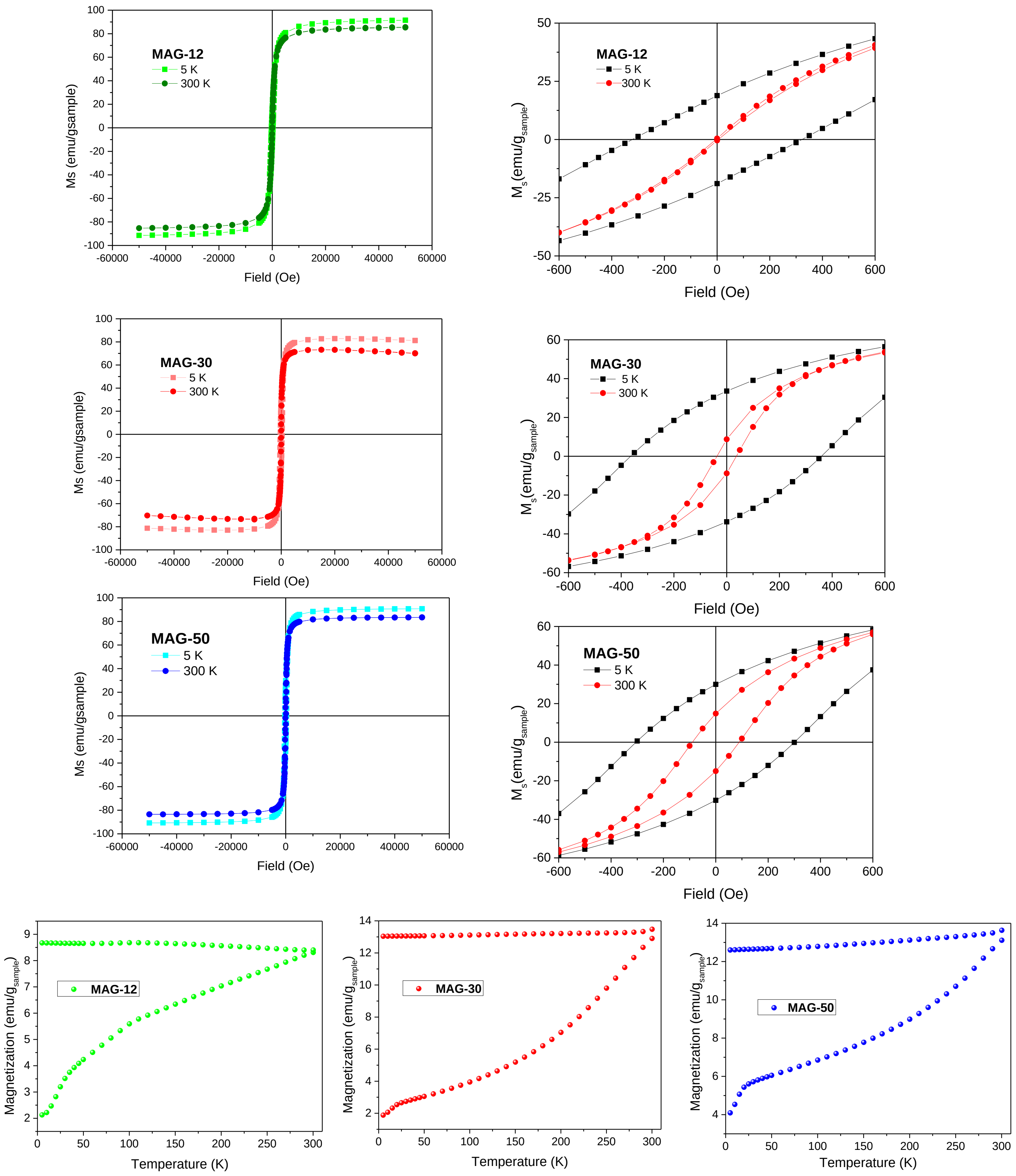

Fig.S3. SQUID measurements (HC and ZFCFC at 100 Oe) of the samples in powder.

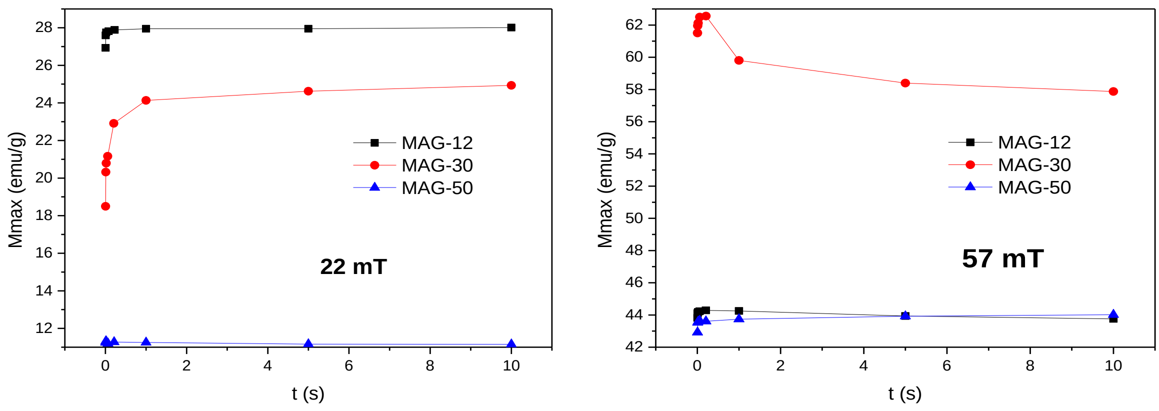

Fig. S4. Evolution of the maximum magnetization as a function of the measurement time for applied fields of 22 mT and 57 mT.

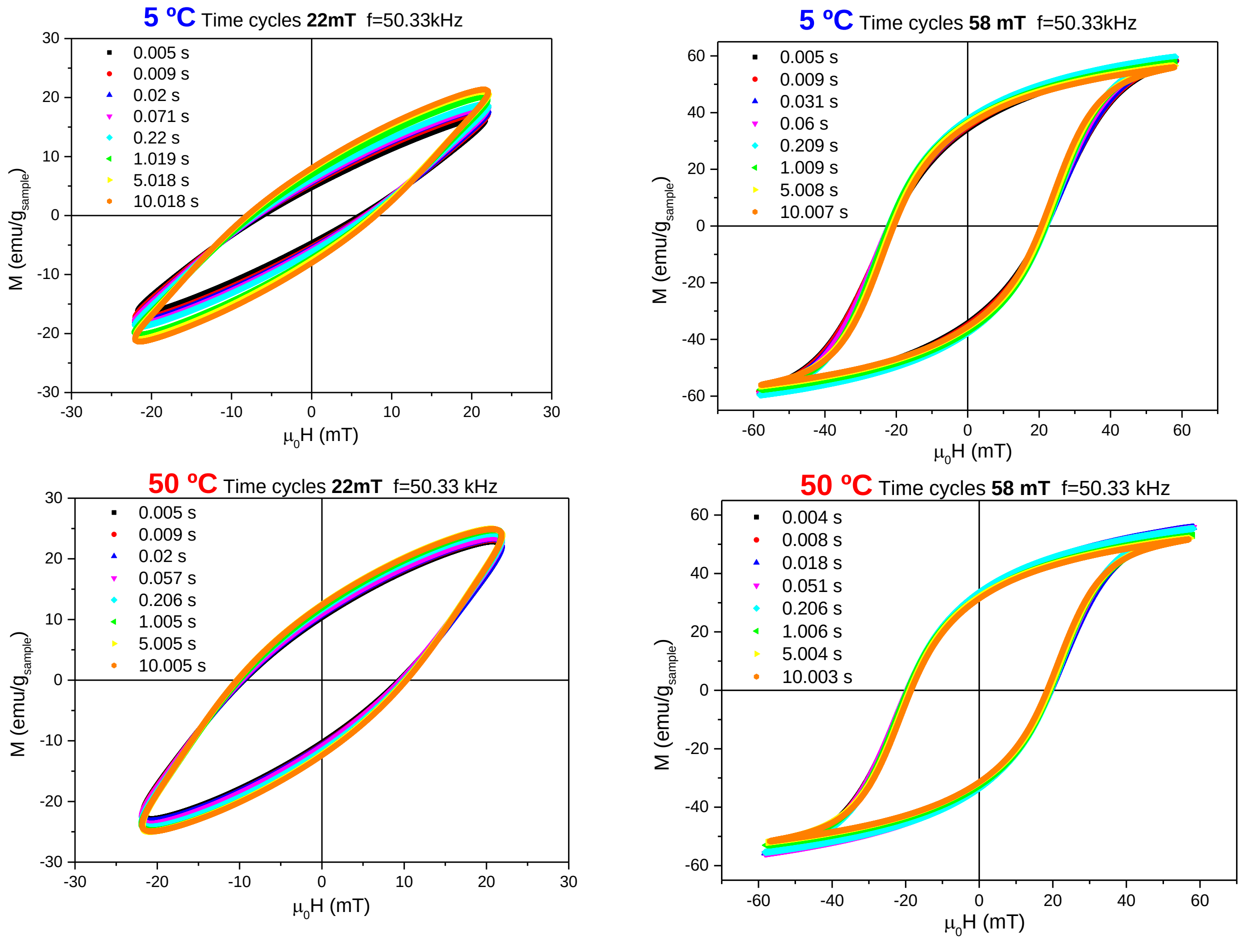

Fig. S5. Evolution of the hysteresis cycles as a function of the measurement time for sample MAG-30 at two different initial temperatures at 22 mT and 58 mT.

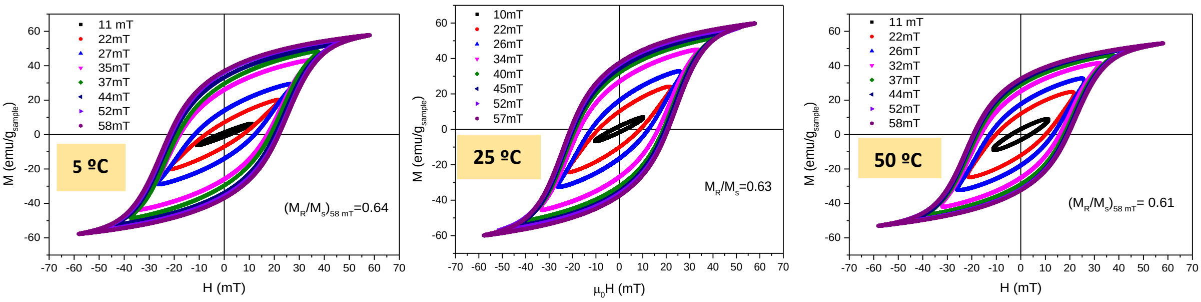

Figure S6.Evolution of MAG-30 high frequency hysteresis loops as a function of the field for different initial temperatures (5, 25 and 50 °C). t_{meas}=1 s. Frequency: 50.33 kHz.

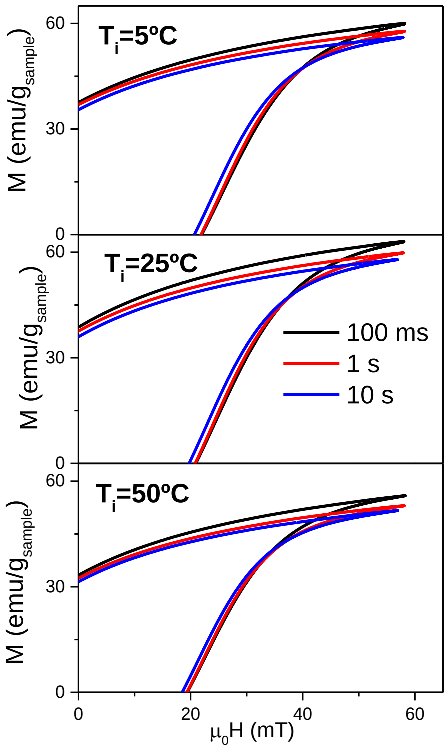

Fig. S7. Hysteresis cycles at 57 mT at three different initial temperatures and measured at different measurement times.

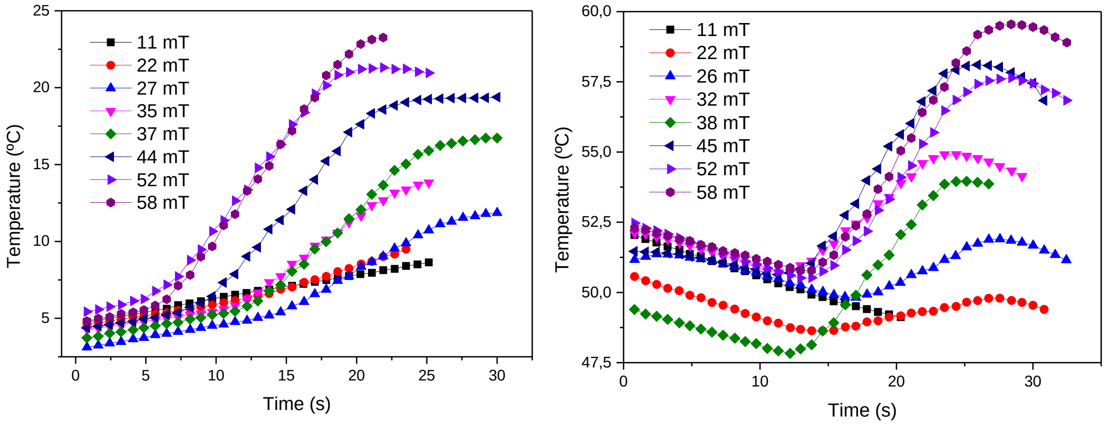

Fig. S8. Temperature curves recorded simultaneously with the AC loops for sample MAG-30 starting from different initial temperatures when applying fields ranging from 10 to 60 mT and a frequency of 50.33 kHz.

Table S1. Neel and Brown relaxation times at zero field assuming $K=10^4 J/m^3$. The values in parentheses correspond to the peak values.

SAMPLE	R_{TEM} (nm)	V_{TEM} (m^3)	τ_N (s)	R_{DLS} (nm)	τ_B (s)
**MAG-12**	3-10 (6)	10^{-25}-$4.2\cdot10^{-24}$ ($9\cdot10^{-25}$)	$1.3\cdot10^{-9}$-$2\cdot10^{-5}$ ($2.6\cdot10^{-8}$)	60	$7\cdot10^{-4}$
**MAG-30**	10-25 (16)	$4\cdot10^{-24}$-$6\cdot10^{-23}$ ($2\cdot10^{-23}$)	$2\cdot10^{-5}$-$8\cdot10^{53}$ ($6.7\cdot10^{8}$)	73	**10^{-3}**
**MAG-50**	17-35 (26)	$2\cdot10^{-23}$-$2\cdot10^{-22}$ ($7.4\cdot10^{-23}$)	$9.3\cdot10^{11}$- $5\cdot10^{200}$ ($3.9\cdot10^{68}$)	1300	**10**
